# Use of Thermistor Temperature Sensors for Cyber-Physical System Security

**DOI:** 10.3390/s19183905

**Published:** 2019-09-10

**Authors:** Carson Labrado, Himanshu Thapliyal, Stacy Prowell, Teja Kuruganti

**Affiliations:** 1 Department of Electrical and Computer Engineering, University of Kentucky, Lexington, KY 40506, USA; 2 Cyber and Applied Data Analytics Division, Oak Ridge National Laboratory, Oak Ridge, TN 37831, USA; 3 Computational Sciences and Engineering Division, Oak Ridge National Laboratory, Oak Ridge, TN 37831, USA

**Keywords:** Physically Unclonable Function (PUF), sensor PUF, cyber-physical systems, Internet of Things (IoT), thermistor, security

## Abstract

The last few decades have seen a large proliferation in the prevalence of cyber-physical systems. This has been especially highlighted by the explosive growth in the number of Internet of Things (IoT) devices. Unfortunately, the increasing prevalence of these devices has begun to draw the attention of malicious entities which exploit them for their own gain. What makes these devices especially attractive is the various resource constraints present in these devices that make it difficult to add standard security features. Therefore, one intriguing research direction is creating security solutions out of already present components such as sensors. Physically Unclonable Functions (PUFs) are one potential solution that use intrinsic variations of the device manufacturing process for provisioning security. In this work, we propose a novel weak PUF design using thermistor temperature sensors. Our design uses the differences in resistance variation between thermistors in response to temperature change. To generate a PUF that is reliable across a range of temperatures, we use a response-generation algorithm that helps mitigate the effects of temperature variation on the thermistors. We tested the performance of our proposed design across a range of environmental operating conditions. From this we were able to evaluate the reliability of the proposed PUF with respect to variations in temperature and humidity. We also evaluated the PUF’s uniqueness using Monte Carlo simulations.

## 1. Introduction

Internet of Things (IoT) devices are known to contain more security risks than conventional computing devices [1]. IoT devices can contain a multitude of vulnerabilities including insecure access interfaces, deployment locations that allow for easy unprotected physical access, and insufficient cryptographic mechanisms (including none at all in some cases) [1,2,3]. This is especially concerning when coupled with that fact that there are currently (as of 2018) 7 billion actively connected IoT devices (39.3% of all connected devices worldwide). These numbers are projected to grow to the point that in 2025 there will be 21.5 billion actively connected IoT devices worldwide. IoT devices would then represent 62.5% of all actively connected devices [4].

Attackers have already shown that they are more than willing to make IoT devices the focus of their attacks. In the last few years for example, compromised IoT devices have been used to create botnets. Botnets are a network of compromised machines that an attack can be used for a variety of malicious purposes including distributed denial-of-service (DDoS) attacks, password cracking, and cryptocurrency mining. Once a machine is infected, it seeks to propagate the infection to other machines in its network by exploiting known vulnerabilities [1]. IoT devices would appear to present an ideal target due to a combination of their lack of security features and the sheer number of these devices that are currently in existence. The Mirai botnet in late 2016 was the first major botnet to be primarily composed of embedded and IoT devices. At its peak the botnet had infected 600 thousand devices [5]. One DDoS attack launched by the botnet was able to disrupt service to many prominent websites including Twitter, New York Times, Reddit, and Airbnb by targeting Domain Name Service (DNS) company Dyn [6]. A separate DDoS attack against French webhost OVH set the record for largest recorded DDoS attack with a size of at least 1.1 terabits per second (tbps) [7].

The threats faced by IoT devices are just one, albeit very notable, example of the security threats that are facing cyber-physical systems as a whole. Other cyber-physical systems such as industrial control systems (ICS), smart grid, medical devices, and smart cars have been shown to be similarly vulnerable to attackers [8]. Some examples include denial-of-service (DoS) attacks against ICS [9] and smart cars [10] and exploiting the lack of encryption in medical devices [11] and smart grids [12].

As originally designed, the dominant methods of security in these systems were related to “security by obscurity”. Devices were assumed to operate in isolation, and it was therefore difficult for an attacker to access them. However, the push towards a connected world has resulted in many of these previously isolated devices now including support for external communication over a variety of networks. The increase in connectivity has also introduced several previously unconsidered possible attack vectors.

On the surface, just simply introducing more security features to these devices seems to be a reasonable approach to protect them from attackers. Unfortunately, these devices commonly have low power, small amounts of available memory, and limited processing capabilities. These factors can prove prohibitive to adding new security features; as a result, researchers have begun to explore nonstandard solutions. Physically Unclonable Functions (PUFs) are one such area that has drawn interest. PUFs are a class of device that are physical implementations of a function. PUF designs leverage their own intrinsic variations so that each copy of the PUF will have its own unique operation in the form of generating outputs that are unique to that copy. Previous works have shown how PUFs could be used to securely generate and store secret keys [13,14] while other works have proposed PUF-based security protocols for use in protecting sensor nodes [15] or securing radio-frequency-identification (RFID) systems [16].

While PUFs could prove to be a novel security solution, their integration would not be completely seamless as it could introduce additional costs such as the monetary cost for the actual hardware or the performance cost of having to operate and/or communicate with the PUF. Therefore, a PUF designed for use in cyber-physical systems should also give special consideration towards reducing these costs as much as possible.

A common functionality in many cyber-physical systems is the ability to monitor physical entities such as temperature, humidity, pressure, luminosity, etc. This can range from being a core function such as a sensor node in an IoT network [17] to being tangential such as a Home Energy Management System (HEMS) that must monitor temperature in order to reduce energy consumption by efficiently controlling a home’s heating and cooling systems [18]. The ability to monitor such a wide range of physical entities is thanks to a similarly wide range of sensors. Thermistor temperature sensors are one example of a popular sensor. In 2017, the thermistor market was valued at USD 74 million. That number is projected to increase to USD 95 million by 2023 [19].

Intrinsic variations are known to exist across a wide range of devices and components and many types of PUFs have already been designed from these materials. The authors believe that creating a PUF from components that are already commonly found in cyber-physical systems could improve the viability of integrating PUFs with these devices. This type of PUF would present a way to add security features without also incurring substantial overhead costs in the form of new hardware. New copies could be created using entirely off the shelf components, circumventing the specialized manufacturing that is required for silicon-based PUF designs which rely on transistor-level variations.

In the existing literature, piezo sensors have been used to create a weak PUF design. However, the use of piezo sensors required including an AC voltage source in the design which further harms its utility. In this work we propose a new design using thermistor temperature sensors to address the shortcomings in the piezo sensor PUF design. We chose to use thermistor temperature sensors due to their widespread appeal as shown by the presence of temperature sensing capabilities in a wide range of fields including health care [20], agriculture [21], and smart home environments [22]. We evaluated the viability of using thermistors as a basis for creating a PUF by testing copies of the proposed design in terms of reliability and uniformity and used Monte Carlo simulations to evaluate the uniqueness.

### Contribution of Work

In this work we propose a methodology that allows for using thermistor temperature sensors to create a PUF that is specifically targeted for application in cyber-physical systems. Our proposed design uses a microcontroller and thermistors which are themselves commonly used by these types of devices. We provide the following:

- Proposal of a PUF circuit design methodology based on intrinsic variations between thermistors.
- Testing the proposed PUF’s reliability over a span of ten days.
- Testing the proposed PUF’s uniformity over a span of ten days.
- Testing the proposed PUF’s reliability over a temperature range of −20 °C to 80 °C.
- Testing the proposed PUF’s reliability over a relative humidity range of 30% to 100%
- Calculating the proposed PUF’s uniqueness through Monte Carlo simulations on 1000 simulated instances.

The rest of this paper is organized as follows: Section 2 covers PUFs including security applications and design approaches that are relevant to our proposed design; Section 3 describes the design methodology behind our proposed PUF; Section 4 describes the tests used to evaluate our proposed PUF and presents the results of those tests; Section 5 compares our proposed PUF to existing sensor-based PUF designs; and finally, Section 6 concludes the paper by providing a summary of our results.

## 2. Background and Related Work

This section provides information on PUFs including different design approaches and examples of their usage in security applications.

### 2.1. Physically Unclonable Functions

PUFs are a type of device that are commonly used in security applications. PUFs take a given “challenge” or input and use it to produce an associated “response” or output. A challenge and its associated response are collectively referred to as a challenge-response pair (CRP). PUFs are especially designed in a way that make them impossible to clone, hence the name “physically uncloneable”. PUF operations rely on their own intrinsic variations that are commonly introduced during the manufacturing process. These variations are random and result in each instance of a given PUF with unique CRPs.

**Figure 1:**
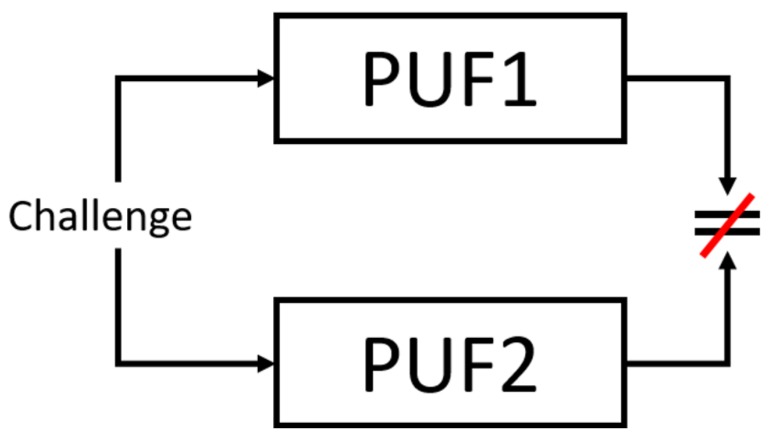
Uniqueness Property of PUF.

Figure 1 shows an example of the uniqueness property of PUFs. In the figure, there exists two copies of the same PUF: PUF1 and PUF2. Each PUF copy is supplied with an identical challenge. The PUFs will then produce their own unique responses for a given challenge. The end result is despite being supplied the same challenge, PUF1 and PUF2 generate responses that are not equivalent.

Additionally, PUFs can be characterized as either “weak” or “strong”. Weak PUFs are characterized as having a very limited number of challenge-response pairs (CRPs), typically just one. They are used in applications where attackers are assumed to not be able to access the responses as knowing just one CRP could be enough to compromise it. Conversely, strong PUFs have a very large number of CRPs. This allows them to be used in applications where an attacker could obtain access to some of the CRPs. This is because strong PUFs should have enough possible challenges that an attacker will not be able to determine all possible CRPs if given a subset of CRPs.

### 2.2. Use of PUF as a Security Measure

The intrinsic properties of PUFs make them well suited to a variety of security applications. Each instance of a PUF should be both unique and unclonable. This places an extra hurdle in the way of attackers that forces them to obtain the actual PUF that is being targeted in the attack as it should be impossible for them to create an exact copy of the PUF. Researchers have begun proposing a wide range of security measures that seek to directly leverage the unique features of PUFs.

One major focus of research has been using PUFs as a way of securely generating and storing secret keys [13,14]. The response from a PUF is used as a seed to generate secret keys. For weak PUFs, the response is a master seed from which all generated secret keys are ultimately derived. The downside to this approach is an attacker only must compromise a single CRP or potentially even one of the keys to compromise all the keys generated by the PUF. Using a strong PUF instead provides more security as each key is derived from a different CRP. The CRPs of strong PUFs are unpredictable and therefore even if an attacker compromises some of the CRPs or keys it has generated, the rest are virtually unaffected.

An approach similar to the one used in key generation can be applied to remove the need for secure memory to store secret keys [14]. Compared to normal unsecure memory, secure memory has the downside of having slower access speeds and being more expensive. As previously described, secret keys can be derived from PUF responses. Rather than store the keys in memory, they can instead be regenerated each time they are needed. This means that the only information that must be stored for each key is the challenge and whatever associated helper data required to generate it. This information is useless to an attacker that does not have access to the actual PUF and thus can be stored in normal unsecure memory.

### 2.3. PUF Design Methodologies

Silicon has proven to be a very popular medium for designing PUFs as researchers are able create designs based on transistor-level variations such as the propagation delay between gates [23] or the initial values found in memory when first powered on [24]. For self-contained devices such as IoT nodes, the implementation of these Silicon-based PUFs, especially ones based on propagation delay, would likely require the addition of specialized hardware or only be viable in certain applications. For example, the sensor node security protocol proposed in [15] uses a memory-based PUF created from the Static Random Access Memory (SRAM) found in commercial Bluetooth Low Energy (BLE) modules. Other researchers have begun exploring the feasibility of implementing a Dynamic Random Access Memory (DRAM)-based PUF in the existing memory of a Raspberry Pi B+ [25].

In addition to Silicon, there exists a wide range of components and materials which are suitable for PUF design [26]. The designs of Non-Silicon-based PUFs prove to be much more varied than normal silicon-based designs. Of particular interest are sensor-based PUFs as they are the category of PUF that our proposed design fits into. Comparatively little research exists on sensor-based PUFs. However, sensors and similar sorts of measurement devices are especially attractive for designing PUFs since their core functionality of measuring and reporting values can be directly incorporated into a PUF. Sensor PUF designs have been proposed based on a large range of components including microelectromechanical systems (MEMS)-based sensors [27,28,29], device touchscreens [30], photodiodes [31], solar cells [32], and piezoelectric sensors [33]. More information about these designs will be presented in Section V. For further information about other PUF designs, a number of existing comprehensive literature surveys are available. We point interested readers to any one of the following works: [34,35,36].

## 3. Proposed Design of a Thermistor Temperature Sensor-Based PUF

A thermistor is a temperature sensing device whose resistance changes with temperature. The design of our proposed PUF uses the on the fact that variations introduced during the manufacturing process will cause individual thermistors to have different resistances at a given temperature. These variations are what allow us to ultimately design a PUF capable of generating unique outputs.

In our proposed design we did not include the implementation of error correcting codes. Error correcting codes have already been proposed as a way to improve the reliability of responses by addressing faults such as bit-flip errors [37,38,39]. However, we wish to evaluate the baseline reliability of our proposed design. Adding error correction codes would obscure these values since the actual results would have been influenced by the codes. The addition of error correcting codes are thus a more relevant consideration for future work that would involve creating a production quality PUF from the proof of concept represented in this work.

### 3.1. Basic Circuit Diagram

The EK-TM4C123GXL model Tiva LaunchPad microcontroller we are using does not have a direct way to measure resistance. Instead, the board has a 12-bit analog-to-digital converter (ADC) capable of detecting voltages between 0 V and 3.3 V. For that reason we needed to create a circuit that would allow the changes in a given thermistor temperature sensor’s resistance to manifest as voltage drops.

**Figure 2:**
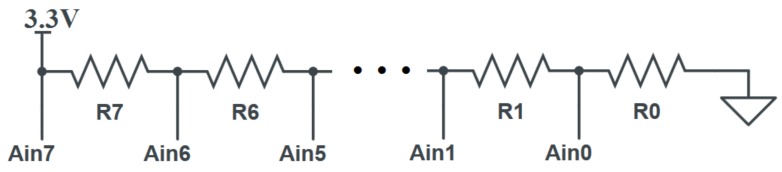
Proposed PUF Circuit Diagram.

Our proposed solution is shown in Figure 2. The thermistors used in our design were NXP KTY81/220. Their operating parameters are shown in Table 1.

The entire circuit consists of 8 thermistor temperature sensors (here represented as resistors *R*) placed in series with a 3.3 V input voltage supplied by the microcontroller. A point before each thermistor is attached to an ADC input pin ($A_{in}$). The microcontroller is then able to take a voltage reading at each point and determine the voltage $V_{R}$ across each thermistor *R* by finding the different between two surrounding points. For example, the voltage across thermistor R5 would be equal to the difference in readings between ADC inputs $A_{in5}$ and $A_{in4}$. The following equations show all the calculations that are made to determine the voltage across each thermistor:

(1) $$\begin{array}{c} \begin{array}{c} V_{R7}=A_{in7}-A_{in6}\\ V_{R6}=A_{in6}-A_{in5}\\ V_{R5}=A_{in5}-A_{in4}\\ V_{R4}=A_{in4}-A_{in3}\\ V_{R3}=A_{in3}-A_{in2}\\ V_{R2}=A_{in2}-A_{in1}\\ V_{R1}=A_{in1}-A_{in0}\\ V_{R0}=A_{in0} \end{array} \end{array}$$

Additionally, singular values read by the ADC can be noisy and slightly vary between readings. As a countermeasure, the final value for each ADC reading is actually the result of taking 100,000 readings and averaging the results.

### 3.2. Complete Architecture

**Figure 3:**
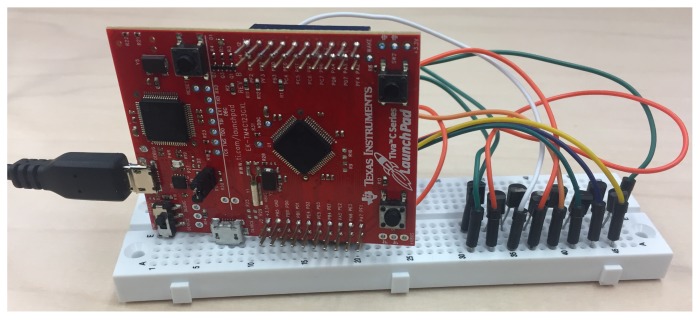
Prototype Implementation of Proposed Thermistor Based PUF.

Our proposed design requires 8 thermistor temperature sensors. Each sensor is connected to a microcontroller in the configuration shown in Figure 2. The onboard ADC is used to sample the voltage readings at each point and uses that data to ultimately derive a voltage drop across each thermistor. After this step is completed, an algorithm can be used to process the individual voltage data and construct a 128-bit response. One such example algorithm can be found in [33]. That algorithm generates a response by making a series of comparisons between total output readings for predetermined groups of a given component. That algorithm assumes that each component should have the same reading, and any differences are solely due to their intrinsic variations. This means that actions such as applying heat to some of the thermistors will result in unreliable readings. The end result is a PUF design that is directly based on thermistor temperature sensors. Figure 3 shows a picture of the fully constructed PUF.

## 4. Testing Configuration and Results

The responses generated from our proposed PUF design were tested to evaluate their reliability and uniformity (as originally described in [41]). In addition, we evaluated the uniqueness of the design by performing Monte Carlo simulations with 1000 simulated copies of the PUF.

### 4.1. Reliability Testing

The reliability of a PUF is a measure of how often it will produce the correct response. The ideal reliability value of a PUF is 100%. This indicates that the PUF will never produce an erroneous response to a given challenge. The following equation (first described in [41]) is used to calculate the reliability for a n-bit response:

(2) $$Reliability=100\%-\frac{1}{m}\sum_{t=1}^{m}\frac{HD(R_{i},R_{i,t}^{{}^{\prime }})}{n}\times 100\%$$

In this equation, $R_{i}$ is a chosen reference response from PUF instance *i*. $R_{i}^{\prime }$ is a response generated under different environmental conditions. A total of *m* responses are collected with different environmental conditions. $HD(R_{i},R_{i,t}^{\prime })$ is the hamming distance (HD) between the reference response ($R_{i}$) and the *t*-th generated response ($R_{i,t}^{\prime }$).

**Figure 4:**
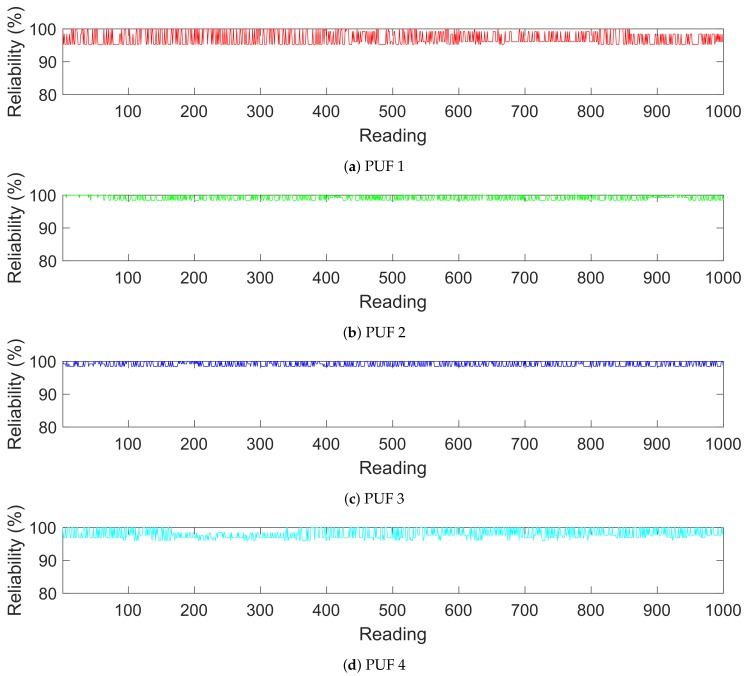

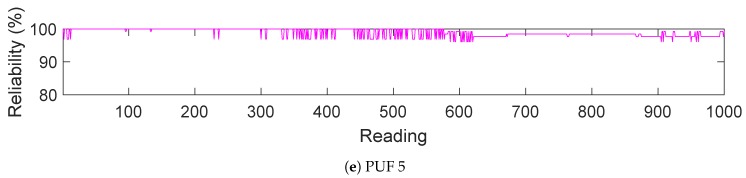
Reliability of PUFs Against Repeated Response Generation.

For our initial reliability testing we took 1000 consecutive readings from 5 copies of our proposed PUF. The first response generated by each PUF was used as the reference response. All readings were taken in a lab space under normal room conditions. Figure 4 shows the graphs for the reliability values of the responses generated by each PUF. The graphs show that each PUF copy maintains a level of reliability that remains close to the ideal value of 100%. Table 2 contains the average reliability values for each copy of the PUF. Among the five copies of the proposed PUF, PUF2 had the highest average reliability at 99.16% while PUF1 had the lowest at 97.09%. The overall combined average reliability for the tested copies was 98.46%.

#### 4.1.1. Temperature Reliability Testing

**Figure 5:**
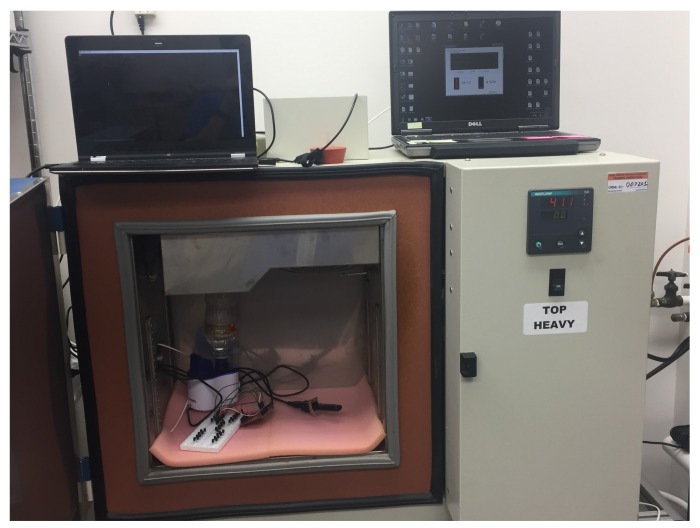
Testing Chamber.

**Figure 6:**
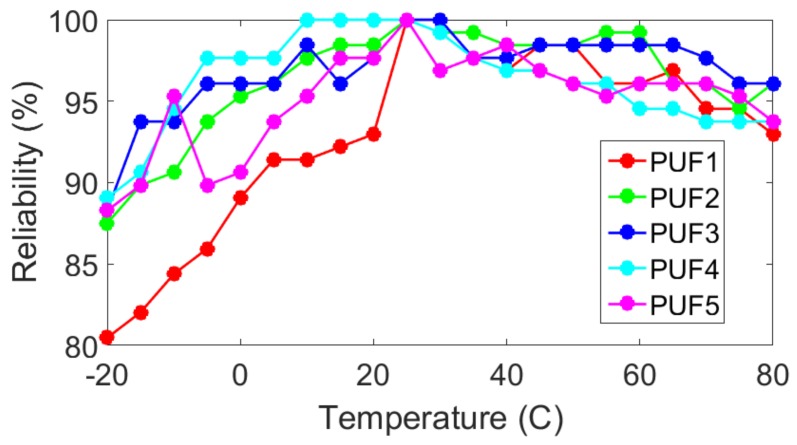
Reliability with Respect to Temperature. 25 °C was used as the reference value and the measured range was −20 °C to 80 °C in increments of 5 °C.

The next phase of reliability testing involved taking readings on each PUF over a range of −20 °C to 80 °C in increments of 5 °C. This was achieved by using the temperature chamber shown in Figure 5 and the graph of the results is shown in Figure 6.

25 °C was used as the reference temperature for determining the reliability values. This is why each copy of the PUF shows 100% reliability at 25 °C. The graph shows that the reliability values begin to fall off as the temperature moves away from the reference temperature of 25 °C. It is worth noting that PUF1 had a more pronounced decline than the other copies of the PUF did as the temperature moved towards −20°. This could be due to just random chance as the other 4 copies of the PUF remain relatively close together. In addition, PUF1 does not suffer a similarly drastic fall in reliability compared to the other copies of the PUF as the temperature approaches 80°. Even though PUF1’s average reliability was a relatively respectable 92.97%, it was still the lowest average reliability among the tested PUFs. Table 3 shows the average reliability for each copy of the PUF. The overall total average reliability for the set was 95.49%.

#### 4.1.2. Relative Humidity Reliability Testing

**Figure 7:**
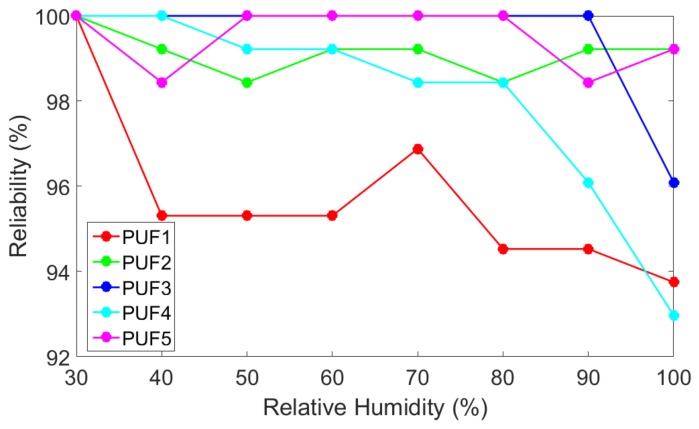
Reliability with Respect to Relative Humidity. 30% was used as the reference value and the measured range was 30% to 100%.

Reliability testing was also performed with respect to relative humidity. 30% relative humidity was used as the reference values and the relative humidity increased from 30% to 100% in increments of 10%. Figure 7 shows the reliability of the PUFs as the relative humidity increases from 30%. Overall, the PUFs seemed to be resistant to changes in relative humidity. Most copies did not show consistent drops in reliability until the relative humidity reached 80%. Table 4 shows the average reliability for each copy of the PUF. PUF1 once again demonstrated the lowest reliability of the test group with an average reliability of 95.70%. The overall total average reliability was 98.30%.

### 4.2. Uniformity Testing

The uniformity of a PUF describes how “balanced” its responses are, i.e., what is the prevalence of 1’s vs. 0’s in the bits of the responses. Ideally, there will be an equal number of 1’s and 0’s to maximize the difficulty for an attacker trying to guess the value of a given bit. This ideal scenario is represented by a uniformity value of 50%. The following equation (first described in [41]) is used to calculate the uniformity of a n-bit response:

(3) $$Uniformity=\frac{1}{n}\sum_{l=1}^{n}r_{i,l}\times 100\%$$

In the above equation, $r_{i,l}$ represents the *l*-th bit of a *n*-bit long response generated by PUF instance *i*. In order to obtain a general uniformity of PUF we averaged together all the readings for a given test. Table 5 shows the average uniformity value for each copy of the proposed PUF across each of the areas of testing (1000 consecutive responses, temperature, and humidity). The overall average uniformity values for the different tests were 50.22%, 49.34%, and 47.91%, respectively. On average, the uniformity values were very close to the ideal value of 50%.

### 4.3. Uniqueness Testing

As described in [41], the uniqueness of a PUF represents the ability to distinguish one particular instance of a PUF from a group of PUFs of the same type. The ideal uniqueness value is 50%. The following equation is used to calculate uniqueness:

(4) $$Uniqueness=\frac{2}{k(k-1)}\sum_{i=1}^{k-1}\sum_{j=i+1}^{k}\frac{HD(R_{i},R_{j})}{n}\times 100\%$$

The above equation determines the average hamming distance (HD) among *k* total PUFs. $R_{i}$ and $R_{j}$ represent *m*-bit responses produced by PUFs *i* and *j*, respectively where i≠j.

The common method for evaluating the uniqueness property of a PUF is by performing Monte Carlo simulations as this allows many unique copies to be generated. For our simulations we created 1000 simulated copies of the PUF. We first created a normal distribution of resistors using the manufacturer specified resistances at 25 °C [40]: minimum of 1960 Ω, maximum of 2040 Ω, and typical of 2000 Ω. Each simulated instance was created by randomly choosing 8 resistors from the distribution. The uniqueness was determined to be 49.89%.

## 5. Comparison to Existing Designs

It should be noted that other PUF designs which are effectively based on measuring differences in resistance values have been proposed. Those designs are based on materials such as magnetoresistive RAM (MRAM) [42], memristors [43,44], and on-chip transistors [45] and metal wires [45,46]. These designs share a common theme with our proposed thermistor PUF of using unique resistances to produce a response. However, we do not feel this is strong enough of a justification to include these designs in direct comparisons that we will do with other sensor PUF designs. The main reason is that one of the goals in creating sensor PUFs is that theoretically a device that already contains the requisite number of sensors could function as a PUF without needing to add any additional hardware. Much like Silicon PUFs, these resistance-measuring PUFs would have to be specifically added to the target device. Furthermore, the resistances of thermistors are designed to change with temperature and can therefore be more sensitive than the components in other designs. Variations in physical properties due to temperature are not intended to be the core operating mechanic of those designs (e.g., allowing thermistors to measure temperature). Sensors on the other hand are generally designed to change one of their physical properties in a significant and predictable way as a direct response to the environmental condition they are monitoring. That same physical property also serves as the basis for creating a sensor PUF from a given sensor. It is for these reasons the focus of our comparisons will be PUF designs that are based on sensors.

Certain difficulties were encountered when attempting to compare the results of our proposed thermistor PUF to existing sensor PUF designs. Unfortunately, sensor PUFs are less popular than Silicon-based PUFs and thus there is comparatively little directly applicable existing research for which we can compare our work. This is further exacerbated by the fact that works that have proposed sensor-based designs do not tend to include performance metrics that can be directly compared with our results. Among the existing sensor-based PUF designs, we are only able to make a direct comparison of performance metrics with the piezo sensor-based design [33]. Comparisons to other designs will solely focus on the functional aspects of the PUF designs.

The devices that we will specifically highlight are microelectromechanical systems (MEMs)-based sensors [27,28,29], device touchscreens [30], photodiodes [31], solar cells [32], and piezo sensors [33]. These designs have certain drawbacks that could hinder their adoption by cyber-physical systems. The piezo sensor PUF [33] requires a sinusoidal input source which is not always readily available certain devices. Additionally, piezo sensors cannot be considered to be prevalent as thermistor temperature sensors since vibration sensing is less common when compared to temperature sensing. The MEMs gyroscope designs [27,28] generate responses based on the output of a MEMs gyroscope. The major concern would be how easily a given gyroscope orientation could be reproduced by a user. A different MEMs-based approach is a ring oscillator (RO) PUF design in which the ring oscillators are constructed from pressure sensing MEMs relays [29]. This design is costly as it requires a separate RO for each bit in the response in addition to bias generation circuitry to control the relays. The touchscreen design [30] is subject to the same type of concern. The design generates a response based on a user’s ability to trace a specified pattern on the screen. There should be a certain amount of variance in results every time a user attempts to replicate the same fine movements that would be used to trace a specified pattern. The photodiode-based design [31] is subject to a sort of chicken and egg problem where its design actually requires a conventional PUF to operate. Lastly, the solar cell work [32] shows that solar cells could potentially be used as a PUF, but stops short of proposing a complete design. Table 6 contains a summary of the drawbacks of various sensor PUF designs.

Our proposed design does not suffer from any of the previously mentioned drawbacks that are present in existing designs. One potential concern is the number of thermistors required to implement our proposed design will not always be present in a given cyber-physical device or system. However, some areas such as certain industrial applications [47,48] which make use of redundant temperature sensors could be especially suitable thanks to the larger than normal number of temperature sensors.

In terms of actual performance metrics, we were only able to make direct comparisons with the reliability and uniformity results between our proposed design and those from the piezo sensor-based PUF [33]. Uniqueness values were not reported. Table 7 contains these values for both our proposed design and the previous piezo sensor work. The average reliability and uniformity across three copies of the piezo PUF was calculated to be 96.07% and 47.24%, respectively. Our proposed design had an average reliability of 98.46% and an average uniformity of 50.22%.

This improvement could be attributed to a couple of factors. The first possibility is the circuit used by our proposed PUF could be more conducive to producing consistent responses. The piezo design required using an ADC to sample AC waveforms which could introduce noise into the measurements. The fact that our proposed design samples what should be steady DC voltages means that the overall sampling process is more straightforward and thus more consistent. A second possible contributing factor is some unspecified aspect of the physical properties of thermistor temperature sensors could simply make them better suited than piezo sensors for constructing PUFs.

A direct comparison of reliability with respect to temperature is complicated by the testing method employed for the piezo PUF. Both our proposed PUF and the piezo PUF used 25 °C as a reference temperature. However, two different chambers were used to test ranges of −20 °C to 0 °C and 25 °C and 80 °C with the range of 0 °C to 25 °C being extrapolated. This prevents a direct comparison in terms of average reliability values. What can be noted is the reliability for the piezo PUF faces a much sharper drop in reliability (below roughly 88%) than any of the thermistor PUFs in which the lowest recorded reliability was 92.97% at 80 °C for PUF1. Additionally, for the range of −20 °C to 0 °C the piezo PUF had its reliability generally drop as the temperature approached 0 °C. Its reliability at −20 °C was better than all the tested copies of our proposed thermistor temperature sensor PUF. However, its reliability at 0 °C was worse than any of the copies of our proposed thermistor temperature sensor PUF.

## 6. Discussion & Conclusions

In this work, we have proposed a novel PUF design for use in cyber-physical systems by using thermistors which are components commonly found within the field. The actual design uses a microcontroller to compare the summed voltage outputs across predetermined groups of thermistor temperature sensors to generate a weak response. Monte Carlo simulations produced a uniqueness value of 49.89% which is very close to the ideal value of 50%. Our proposed design was shown to have improved overall reliability and with regards to changes in temperature when compared to the existing design based on piezo sensors [33]. Additional reliability testing with respect to relative humidity appeared to show that the proposed design is relatively unaffected by humidity values less than 80%. As a future work, the addition of error correcting codes could help improve the reliability values of the base design.

It is worth noting that this design should be treated as a proof of concept and not a fully realized security solution. The main goal in creating this device was to conduct a preliminary exploration to determine if thermistor temperature sensors are a viable option for PUF creation when compared to existing sensor-based PUF designs. The prototypes we created for testing purposes were meant to only address this question of viability. The prototypes are vulnerable to physical attacks such as an attacker manually measuring the voltage drops across each thermistor and then creating a model of the PUF. Other researchers have already explored mitigation methods such as implementing tamper-resistance [49,50] and providing protection from side channel attacks [51,52,53]. Exploring the integration of existing solutions or devising new concepts are outside of the scope of this paper and should instead be considered to be avenues for future work when designing a full-scale production quality implementation.

## Figures and Tables

**Table 1:** Operating Parameters of NXP KRY81/220 Temperature Sensors [40].

Parameter	Value
Operating Temperature	−55 °C to 150 °C
Typical Resistance @ −20 °C	1367 Ω
Typical Resistance @ 25 °C	2000 Ω
Typical Resistance @ 80 °C	2980 Ω

**Table 2:** Average Reliability Values of Proposed PUF Instances when Generating 1000 Consecutive Responses.

PUF1	PUF2	PUF3	PUF4	PUF5	Total
97.09%	99.16%	99.09%	98.08%	98.91%	98.46%

**Table 3:** Average Reliability from −20 °C to 80 °C.

PUF1	PUF2	PUF3	PUF4	PUF5	Total
92.97%	96.32%	96.84%	96.21%	95.09%	95.49%

**Table 4:** Average Reliability from 30% to 100% Relative Humidity.

PUF1	PUF2	PUF3	PUF4	PUF5	Total
95.70%	99.12%	99.12%	98.05%	99.51%	98.30%

**Table 5:** Average Uniformity Values of Proposed PUF Instances.

	PUF1	PUF2	PUF3	PUF4	PUF5	Total
Consecutive	49.66%	49.96%	50.05%	49.48%	51.94%	50.22%
Temperature	48.59%	48.21%	49.52%	48.92%	51.45%	49.34%
Humidity	47.46%	46.58%	49.51%	47.85%	48.14%	47.91%

**Table 6:** PUF Comparison.

PUF	Description	Drawback
Piezo [33]	Compares summations of voltage drops across groups of piezo sensors	Requires an additional AC input voltage. Limited applications compared to proposed design.
MEMs Gyro [27,28]	Responses are derived from the output of a MEMs gyroscope	Concerns about being able to repeatedly produce a desired CRP.
MEMs Pressure [29]	Ring Oscillator (RO) design using pressure sensitive MEMs relays.	Significant overhead due to additional circuitry.
Touchscreen [30]	A user traces a specified pattern displayed on the touchscreen	Concerns about being able to repeatedly produce a desired CRP.
Photodiode [31]	Compares summation of sensor groups based on the output of a PUF	Correct operation requires an existing conventional PUF.
Solar Cells [32]	Testing results show that solar cells produce unique voltages for the same light source	Complete design not proposed.
Proposed Design	Uses microcontroller to compare readings from groups of thermistor temperature sensors to generate a weak response.	Requires more thermistor temperature sensors than may already exist in certain systems.

**Table 7:** PUF Comparison.

	Piezo [33]	Proposed
Uniformity	47.24%	**50.22**%
Reliability	96.07%	**98.46**%
